# Trial forge: a systematic approach to making trials more efficient

**DOI:** 10.1186/1745-6215-14-S1-O121

**Published:** 2013-11-29

**Authors:** Shaun Treweek

**Affiliations:** 1 University of Aberdeen, Aberdeen, UK

## Aim

1) To raise awareness of the need to make trials more efficient and 2) To hear delegates’ views on more collaborative trial methodology research.

## Background

Doing a trial can be a challenge but do we make it a greater challenge than it needs to be? For example, is the research question the one most relevant to patients, health professionals and policymakers? Do we really need to collect all those data? Is selecting that site a good idea and why? Do we know our retention strategies are effective and if not, why are we using them without evaluation?

## Changing the status quo

In this talk I’ll suggest that we rethink the way we do trials. We are unlikely to find a magic bullet that will make trials easy. We could, however, make trials more efficient by being more sceptical of what we do and start to systematically identify discrete trial processes, ask ourselves why we do them, collate what is known about the best ways to achieve this and then develop collaborative programs of methodological work to address any knowledge gaps. This, together with effective programs of dissemination to those designing, governing, running and using trials, is more likely to lead to step-changes in trial efficiency than a series of high quality, but uncoordinated, methodological studies.

## Conclusion

There is a tendency to do trials the way we do because that’s the way we do them. We should work together to make our trial processes more evidence-based.

